# Estimating indicator weights for the motor health of pre-school-aged children: an integrated-methods approach

**DOI:** 10.3389/fpubh.2026.1798183

**Published:** 2026-07-27

**Authors:** Dongxu Du, Linyan Chai, Qiang Fu, Gao Yang, Yujiao Huang, Chenhua Huang

**Affiliations:** 1 Sports Academy, Zunyi Normal University, Zunyi, China; 2 Surgical Center for Neurological Diseases, Zunyi First People's Hospital, Zunyi, China; 3 School of History, Culture and Tourism, Zunyi Normal University, Zunyi, China; 4 Department of Physical Education, Guiyang Pre-school Education College, Guiyang, China; 5 School of Education, Central China Normal University, Wuhan, China; 6 College of Childcare Services and Health Care, Guangxi College for Preschool Education, Guangxi, China

**Keywords:** health promotion (HP), indicator weights, integrated methods, motor health, pre-school children

## Abstract

**Introduction:**

Motor health and long-term development of pre-school children is a pressing issue that requires urgent attention. While motor health is critically important for the early growth stage of pre-school children, establishing a multi-dimensional health evaluation indicator weighting system using an integrated subjective-objective weighting method remains a cutting-edge challenge in current research.

**Methods:**

WPS Office 6.4 and IBM^®^ SPSS^®^ Statistics 26.0 were used to analyze the data, with the significance threshold set at *P*-value < 0.05. The integrated subjective-objective weighting method mainly consists of four steps: (i) the Delphi subjective weighting method, including *A*_*i*_, *A*_*s*_, *A*_*a*_, *W*_*i*_, $W_{i}^{\prime }$ω; (ii) the entropy objective weighting method, including *X*_*ij*_, *P*_*ij*_, *E*_*j*_, *d*_*j*_, and *W*_*j*_; (iii) the comprehensive weighting method, including relevant parameters $W_{j}^{*}$, among others; (iv) IBM^®^ SPSS^®^ Amos (TM) 24.0 was used to conduct further data analysis.

**Results:**

The total combined weight of the six primary indicators is 1.0, with individual weights ranging from 0.109 to 0.213. The corresponding weights of the 26 secondary indicators range from 0.014 to 0.215. Regarding the weighting of first-level indicators, no significant correlation was found between the comprehensive weighting method and the Delphi method (*r* = 0.771, *P* = 0.072), nor between the comprehensive weighting method and the entropy weight method (*r* = 0.600, *P* = 0.208). Similarly, no significant correlation was observed between the Delphi method and the entropy weight method (*r* = 0.143, *P* = 0.787). In contrast, for second-level indicators, the comprehensive weighting method demonstrated a strong correlation with both the Delphi method (*r* = 0.917, *P* < 0.001) and the entropy weight method (*r* = 0.558, *P* = 0.003). However, no significant correlation was detected between the Delphi method and the entropy weight method (*r* = 0.268, *P* = 0.185).

**Conclusion:**

By adopting an integrated subjective-objective weighting method, this study establishes evaluation indicator weights based on two foundations: the intrinsic patterns within the data and expert experience. The weights derived through this dual-foundation approach therefore exhibit improved rationality and credibility, providing important reference value for the comprehensive assessment of pre-school children's motor health.

## 1 Introduction

Motor health and future development of pre-school children are urgent issues to address. Early childhood development refers to the cognitive, physical, linguistic, motor, social, and emotional development of children from 0 to 8 years of age (1). Chinese pre-schoolers exhibit a high prevalence of overweight and obesity, alongside poor sleep and physical activity habits (2). The pre-school years represent a critical period for learning and cognitive development, and physical activity is beneficial for children's physical and mental health (3). According to the WHO's guidelines on 24-h physical activity, sedentary behavior, and sleep for children under 5 years, physical inactivity is a leading risk factor for global mortality and a key contributor to the rise in overweight and obesity (4). Early childhood (under age 5) is characterized by rapid physical and cognitive growth, during which lifelong habits are formed and family routines can be positively shaped. Globally, at least one-third of children under five show signs of stunting, wasting, or overweight; the triple burden of malnutrition—undernutrition, micronutrient deficiencies, and overweight—threatens the survival, growth, and development of children and adolescents, as well as economic progress and national development (5). Improving the quality of the childcare environment has been shown to enhance physical and mental health and promote physical activity among pre-schoolers (6–8).

Motor competence is recognized as a fundamental component of child development and is closely linked to long-term health outcomes. In this study, motor competence in pre-school-aged children is operationalized through key physical capacities: strength, speed, flexibility, coordination, and balance. These attributes serve as measurable indicators, underscoring the role of motor competence in fostering healthy development and mitigating obesity risks (9). Evidence suggests that motor competence positively influences various health indicators in children (10). Physical activity, in turn, demonstrates significant associations with improvements in both physical and psychological wellbeing (11, 12). Motor development is multifactorially determined, shaped by physiological, behavioral, emotional, and environmental factors (13)—a perspective consistent with the integrative framework proposed by Du et al. (14). Furthermore, social and emotional competencies are consistently associated with positive outcomes, including better academic performance, daily living skills, friendship formation, and prosocial behavior (15, 16). Environmental and social factors also play a crucial role in shaping children's mental health (17). Therefore, the pre-school period constitutes a critical developmental window across multiple domains. Motor health during this stage arises from the complex interplay of motor competence, physical health, psychological wellbeing, social-emotional competencies, health-related behaviors, and environmental influences. Given the heightened vulnerability of this age group, early identification of developmental delays, timely implementation of integrated and targeted interventions, and sustained promotion of overall health are essential strategies for optimizing developmental trajectories.

Although prior research has acknowledged the multidimensional nature of motor health in pre-school children and has assessed certain dimensions using either subjective or objective methods, no study to date has integrated core domains—such as motor ability, physical health, mental health, movement-conducive environments, and movement-related behaviors—into a unified assessment framework underpinned by an explicit multidimensional theoretical model. Moreover, no research has yet applied a combined subjective-objective weighting approach to systematically analyze the relative importance of these dimensions and their constituent indicators, thereby establishing a weighting system that is both theoretically coherent and empirically justified. While extensive evidence exists on factors influencing pre-school children's motor health, limitations persist, including fragmented theoretical frameworks, methodological constraints, and limited translational application. Therefore, this study adopts a systematic interdisciplinary approach to construct a theoretically grounded and practically applicable motor health evaluation system for pre-school children, encompassing the development of an indicator system, determination of indicator weights, and establishment of an assessment model.

Given that pre-school children's motor health is a multidimensional and context-sensitive construct, a single methodological approach is inadequate to capture its full complexity. Guided by a pragmatist epistemological stance and interdisciplinary theory, this study integrates expert-based (subjective) judgment with data-driven (objective) information to weight the assessment indicators. A healthy body, positive affect, physical robustness, coordinated movement, sound habits, and foundational life skills are important markers of physical and mental health, as well as pre-requisites for learning and development in other domains. This study builds upon key indicator systems, including motor ability, physical health, mental health, motor health behavior, social-emotional competence, and motor health environment (18), and a multifactorial structural equation model of motor health (14).

Indicator weighting carries both theoretical necessity and practical utility, serving not only to align measurement with theoretical expectations but also to facilitate precise screening, intervention prioritization, and sensitive outcome evaluation. This study aims to construct a weighting system for evaluating motor health in pre-school children. To achieve this, we employ an integrated weighting method combining the Delphi (subjective) and entropy (objective) approaches. The Delphi method, which derives subjective weights through anonymous iterative expert consultation, is widely used across disciplines and helps mitigate cognitive bias (19–21). Similarly, the entropy-based objective weighting method, which captures intrinsic data variability, is extensively applied in diverse fields (22–25). However, reliance on a single weighting method may limit the robustness of the resulting weights. Integrated approaches that combine subjective and objective techniques have therefore gained considerable attention in decision-making and evaluation research. By synergizing expert judgment with data-driven analysis, these hybrid methods enhance the reliability and validity of weight estimation (26, 27).

Drawing on Bronfenbrenner's ecological systems theory, Bandura's social learning theory, the CASEL framework for social-emotional learning, comprehensive evaluation weighting theory, exercise science, and developmental psychology, this study proposes an interdisciplinary approach to construct a pre-school children's motor health evaluation index system (EIS) with strengthened theoretical and practical value. We adopt a comprehensive weighting method that integrates subjective and objective techniques to overcome the limitations of single-method approaches. Through rigorous analysis and validation, this method offers an innovative strategy for indicator weighting, enriching the theoretical foundation in the field of child motor health and providing new research tools and perspectives for future studies.

The innovative contributions of this study are as follows: in response to developmental challenges faced by pre-school children in contemporary society, it is of significant importance to construct a motor health evaluation indicator system and weighting scheme grounded in an interdisciplinary approach. Pre-school children's health should be advanced as a vital component of global health and wellbeing.

## 2 Methods

### 2.1 Participants

This study recruited 14 experts for the Delphi panel through snowball sampling from multiple regions and universities across China. Additionally, 410 pre-school children (201 boys, 209 girls) were selected via random cluster sampling from three districts in Guizhou Province, southwestern China, for motor health assessment. Data collection was strictly conducted in compliance with ethical research requirements. Informed consent and signed documentation were obtained from administrators of relevant Chinese kindergartens, volunteer parents, and participating children. This process rigorously ensured the children's voluntary participation and enforced comprehensive safety protocols. The study protocol was approved by the Ethics Committee of Mahasarakham University (#497-453/2023).

### 2.2 Selection criteria and screening

Delphi panel members were selected through snowball sampling from various regions and universities in China. Inclusion criteria were: (a) employment in the fields of motor training, psychology, physical education, or public health; (b) more than 10 years of work experience; (c) holding an associate senior professional title or above; (d) holding a bachelor's degree or higher; and (e) voluntary participation with positive engagement. Exclusion criteria included: (a) unwillingness to participate for personal reasons; (b) lack of practical experience in motor health-related work; and (c) withdrawal during the study.

Preschool children were selected through random cluster sampling from five kindergartens in Guizhou Province, southwestern China. Inclusion criteria were: (a) typical intellectual development and absence of organic diseases; (b) completion of physical fitness tests according to the National Physical Fitness Test Standards (2023) for early childhood; and (c) informed consent from parents/guardians, with signed consent forms. Exclusion criteria included: (a) refusal or non-cooperation of the child or parent in tests or questionnaires; (b) presence of systemic infectious diseases; (c) congenital or severe chronic conditions such as cardiac, hepatic, or renal insufficiency; and (d) diagnosed neurodevelopmental disorders including cerebral palsy, intellectual disability, autism spectrum disorder, or attention deficit hyperactivity disorder.

### 2.3 Data extraction

Data were collected from January to June 2024. In the first phase, data from expert consultation were obtained to construct the evaluation indicator system and determine subjective indicator weights. In the second phase, pre-school children completed assessments of body composition (height and weight) and motor abilities (grip strength, standing long jump, sit-and-reach, two-footed jump, 15-m obstacle run, and balance beam walk). In the third phase, with the assistance of kindergarten staff, parents of children who had finished the motor ability tests were invited to participate in proxy-reported data collection. Both the expert consultation surveys and parent-proxy questionnaires were distributed via the WeChat^®^ platform.

### 2.4 Data analysis

Data were analyzed using WPS Office 6.4 and IBM^®^ SPSS^®^ Statistics 26.0, with statistical significance set at *P* < 0.05. The integrated subjective-objective weighting method mainly consists of three steps: (i) the Delphi subjective weighting method, including *A*_*i*_, *A*_*s*_, *A*_*a*_, *W*_*i*_, $W_{i}^{\prime }$, ω; (ii) the entropy objective weighting method, including *X*_*ij*_, *P*_*ij*_, *E*_*j*_, *d*_*j*_, and *W*_*j*_; (iii) the comprehensive weighting method, including $W_{j}^{*}$, among others; and (iv) IBM^®^ SPSS^®^ Amos (TM) 24.0 was used to analyze the data.

### 2.5 Research design and methodological flowchart

**Figure 1:**
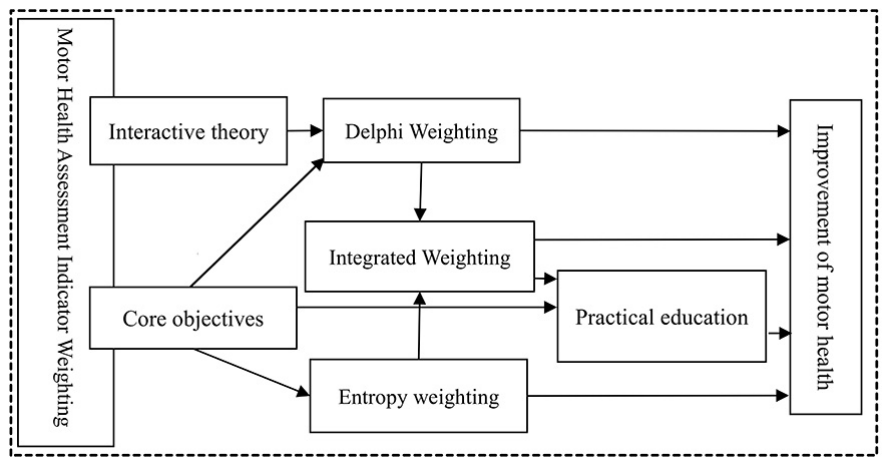
Research design and methodological flowchart.

This study developed an integrated subjective–objective weighting scheme for pre-school children's motor health assessment indicators. The overall research design and methodological flowchart are presented in Figure 1. Grounded in interdisciplinary theory and core research objectives, we synthesized subjective weights derived from the Delphi method and objective weights obtained through entropy analysis into a composite weighting system. This approach shifts the paradigm for advancing children's motor health from a narrow focus on “practical education” to an integrated framework incorporating “interdisciplinary perspectives, ecological environments, and evaluative research.” Accordingly, it promotes the mutual integration of the motor health assessment system and practical education, ultimately contributing to improved motor health outcomes for pre-school children.

### 2.6 Quality control

After two rounds of expert consultation, the Kendall's W coefficient of concordance was 0.803 for primary indicators and 0.758 for secondary indicators, both exceeding 0.7. This indicates good consensus among experts and stable evaluation outcomes, and an expert authority coefficient greater than 0.7 is generally considered acceptable. Before motor ability assessments were conducted, all evaluators received standardized training. During testing, all procedures were strictly implemented in accordance with the operation manual and demonstration videos. Any uncertainties that arose during on-site assessments were clarified by the on-site expert team to ensure data accuracy. The parent-proxy questionnaire was distributed via WeChat^®^ parents were required to complete the questionnaire before they could access their child's motor ability assessment report. They were clearly informed that the questionnaire covered important aspects of children's physical and mental health and was closely linked to the motor assessment results. Furthermore, all scales used in the study demonstrated good internal consistency (Cronbach's α > 0.7) and sampling adequacy (KMO > 0.8), with statistically significant results (*P* < 0.001), which ensured both the validity and reliability of questionnaire responses. The stability and limitations of the weight results were verified through 95% confidence intervals.

## 3 Results

### 3.1 Delphi subjective weighting method for weight estimation

The Delphi method, characterized by anonymity, iterative feedback rounds, and statistical aggregation, is well suited for exploratory fields where clear consensus is lacking. It facilitates the systematic convergence of expert opinions, mitigates individual bias, and yields a preliminary weight vector that reflects collective judgment. The purpose of the Delphi weighting procedure is to obtain relatively objective information, insights, and judgments through repeated independent evaluations by multiple experts, and ultimately reach a consistent and reliable consensus or solution. The Delphi subjective weighting approach is widely applied and has demonstrated sound evaluation outcomes in previous research (28). In this study, an expert consultation questionnaire was developed using a Likert 5-point scale to rate the importance of each evaluation indicator. The research team collected, summarized, and analyzed expert feedback, allowing opinions to converge gradually and resulting in a reliable indicator set and weighting scheme.

After two rounds of expert consultation, the experts' familiarity coefficient, judgment coefficient, and authority coefficient for all evaluation indicators exceeded 0.70. The Kendall's coefficient of concordance (W) was above 0.70, the arithmetic mean of relevance and feasibility scores was ≥4.00, and the coefficient of variation for all items was below 0.25. In the first round, experts received a preliminary indicator list developed from a literature review. They were asked to rate each indicator on relevance, clarity, and importance, and provide open-ended comments on adding, deleting, merging, or modifying indicators. In the second round, after reviewing the distribution of the whole group's responses, experts gave final importance ratings for the revised indicator list, with the aim of reaching consensus on both indicator inclusion and weighting tendencies. During the screening process, indicators were evaluated against three quantitative criteria: full-score ratio, arithmetic mean, and coefficient of variation. When quantitative results were inconsistent, a structured anonymous brainstorming session was held for qualitative discussion, followed by a formal vote to determine whether an indicator should be retained or excluded.

Experts were selected from the fields of sports training, physical fitness assessment, psychology, physical education, school sports, and childhood physical activity. Among the 14 experts who completed the second-round questionnaire, 10 were male and four were female; all held at least a bachelor's degree and the title of associate professor or higher, meeting the basic requirements of their respective professional domains. Seven experts held full professor positions (50%), and seven held associate professor positions (50%). All had over 15 years of work experience. Nine held doctoral degrees (64.3%), three held master's degrees (21.4%), and two held bachelor's degrees (14.3%). Experts engaged in pediatric medicine and educational psychology constituted 42.9% of the sample, while those specializing in physical education and sports training accounted for 57.1%. All 14 experts were familiar with the comprehensive assessment of children's motor health, with 10 (71.4%) being highly familiar. The main computational steps of the Delphi weighting procedure are described below.

#### 3.1.1 Expert scoring and expert authority coefficients

The 14 participating experts scored the relative importance of each evaluation indicator using a 100-point system, with weight ratios assigned in arithmetic progression (e.g., 4:3:2:1).

The authority level of an expert is generally determined by two dimensions: the scientific rigor of their judgment basis, and their familiarity with the evaluation issues. The formula for calculating the degree of authority is displayed in Formula 1:


$$\begin{array}{l} {\text{A}}_{\text{a}}=\left({\text{A}}_{\text{i}}+{\text{A}}_{\text{s}}\right)/2 \end{array}$$ (1)

Where *A*_*a*_ is the coefficient for the expert's authority, *A*_*i*_ is the coefficient for the expert's judgment, and *A*_*s*_ is the coefficient for the expert's familiarity.

In this study, a self-assessment method is adopted, where experts score their familiarity and judgment basis for the corresponding evaluation indicators. Table 1 presents the judgment basis, familiarity, and authority coefficients of each expert.

**Table 1:** Expert judgment, familiarity, authority coefficients.

Experts	Judgment coefficients		Familiarity coefficients		Authority coefficients	
	*A_*i*_*	Ranking	*A_*s*_*	Ranking	*A_*a*_*	Ranking
1	29.8	3	28.8	2	29.3	3
2	23.2	12	21.8	12	22.5	13
3	31.8	1	27.0	6	29.4	2
4	30.2	2	29.6	1	29.9	1
5	27.0	4	27.2	5	27.1	4
6	23.2	11	25.4	8	24.3	9
7	24.4	10	27.6	3	26.0	7
8	23.0	13	24.2	11	23.6	10
9	26.0	6	21.0	13	23.5	11
10	24.8	9	25.2	9	25.0	8
11	25.4	8	19.4	14	22.4	14
12	21.6	14	24.8	10	23.2	12
13	26.6	5	26.2	7	26.4	6
14	25.8	7	27.4	4	26.6	5

Experts' serial numbers are listed in the order in which they completed the questionnaire.

As, the expert familiarity coefficients; Ai, the expert judgment coefficient; Aa, the expert authority coefficients.

#### 3.1.2 Weight calculation of evaluation indicators

The weight calculation formula considering expert authority is presented in Formula 2:


$$\begin{array}{c} {\text{W}}_{\text{i}}={\text{γ}}_{\text{i}1}{\text{A}}_{\text{a}1}+{\text{γ}}_{\text{i}2}{\text{A}}_{\text{a}2}\ldots {\text{γ}}_{\text{im}}{\text{A}}_{\text{am}}/\text{m} \end{array}$$ (2)

In the formula, *W*_*i*_ represents the weighted authority level of the *i*-th indicator, γ_*i*1_γ_*i*2_…γ_*im*_ represents the scoring results of each expert for the *i*-th indicator, *A*_*a*1_*A*_*a*2_…*A*_*am*_ represents the authority level of each expert, and *m* represents the number of evaluating experts.

The weights of the indicators are obtained after normalization. We calculated the normalized weights based on Formula 3:


$$\begin{array}{c} {\text{W}}_{\text{i}}^{\text{′}}={\text{W}}_{\text{i}}/\sum_{\text{i }=\;1}^{\text{m}}{\text{W}}_{\text{i}} \end{array}$$ (3)

In the formula, $W_{i}^{\prime }$ represents the weight of the *i*-th indicator.

#### 3.1.3 Expert coordination coefficient

The Kendall's coefficient of concordance (ω) was calculated to assess agreement among experts Formula 4:


$$\begin{array}{c} \omega =\frac{12}{{\text{m}}^{2}\left({\text{n}}^{3}-\text{n}\right)}\sum_{\text{j }=\;1}^{\text{n}}{\text{d}}_{\text{j}}^{2} \end{array}$$ (4)

In this formula, $\overset{\text{n}}{\underset{\text{j }=\;1}{\sum }}{\text{d}}_{\text{j}}^{2}$ represents the sum of the squared deviations from the mean of the sum of the ranks of all indicators.

The coefficients of concordance for primary and secondary indicators were calculated based on the two consultation rounds. After the second round, ω was 0.803 for primary indicators and 0.758 for secondary indicators, both exceeding 0.70, indicating good consensus among experts and stable evaluation outcomes.

The calculated weights for primary and secondary indicators, as well as the combined weights of secondary indicators, are presented in Table 2. The Delphi weights for primary indicators are: motor ability (0.215), physical health (0.203), mental health (0.188), social and emotional competence (0.127), motor health environment (0.125), and motor health behavior (0.142). The Delphi weights for secondary indicators are: motor ability—speed (0.214), strength (0.176), coordination (0.240), flexibility (0.148), balance (0.222); physical health—body composition (0.479), physiological perception (0.521); mental health—emotional health (0.242), self-esteem (0.213), family contact (0.188), social functioning (0.179), school role (0.179); social and emotional competence—social awareness (0.240), relationship skills (0.218), responsible decision-making (0.186), self-awareness (0.178), self-management (0.178); motor health environment—sports policy (0.178), community environment (0.186), physical training institutions (0.178), school environment (0.218), family environment (0.240); and motor health behavior—sport behavior (0.292), lifestyle (0.265), psychological behavior (0.227), social adaptation (0.216). The combined weights of secondary indicators were computed using the product method, ranging from 0.022 to 0.106; specific values and rankings are provided in Table 2.

**Table 2:** Delphi method indicator weights and combined weights.

Single weight	A: MA	B: PH	C: MH	D: SEC	E: MHE	F: MHB	Composite weight	Ranking
	0.215	0.203	0.188	0.127	0.125	0.142		
A1: Speed	0.214						**0.046**	5
A2: strength	0.176						**0.038**	9
A3: CA	0.240						**0.052**	3
A4: flexibility	0.148						**0.032**	15
A5: balance	0.222						**0.048**	4
B1: body shape		0.479					**0.097**	2
B2: PP		0.521					**0.106**	1
C1: EH			0.242				**0.045**	6
C2: self-esteem			0.213				**0.040**	8
C3: families			0.188				**0.035**	11
C4: SC			0.179				**0.034**	13
C5: schools			0.179				**0.034**	12
D1: SO				0.240			**0.030**	17
D2: RS				0.218			**0.028**	19
D3: DM				0.186			**0.024**	21
D4: SA				0.178			**0.023**	24
D5: SM				0.178			**0.023**	23
E1: SP					0.178		**0.022**	25
E2: CE					0.186		**0.023**	22
E3: SI					0.178		**0.022**	26
E4: SE					0.218		**0.027**	20
E5: FE					0.240		**0.030**	18
F1: MB						0.292	**0.042**	7
F2: lifestyle						0.265	**0.038**	10
F3: PB						0.227	**0.032**	14
F4: adaptation						0.216	**0.031**	16

As, the expert familiarity coefficients; Ai, the expert judgment coefficient; Aa, the expert authority coefficients; MA, motor ability; PH, physical health; MH, mental health; SEC, social and emotional competencies; MHE, motor health environment; MHB, motor health behavior; CA, coordination ability; PP, physiological perception; EH, emotional health; SC, social contacts; SO, social awareness; RS, relationship skills; DM, responsible decision making; SA, self-awareness; SM, self-management; SP, sports policies; CE, community environment; SI, sports institutions; SE, school environment; FE, family environment; MB, motor behavior; PB, psychological behavior. The bolded values represent the combined weight results.

In the Delphi subjective weighting procedure, 95% confidence intervals (CIs) for each weight were estimated using the t-distribution method, as shown in Table 3. Among the primary indicators, motor ability had the narrowest 95% CI [0.182, 0.246], indicating the most concentrated expert opinion, whereas mental health had a relatively wider CI [0.160, 0.225]. The CIs for social-emotional competence, motor health environment, and motor health behavior showed considerable overlap. Among secondary indicators, coordination [0.049, 0.055], balance [0.045, 0.051], and physiological perception [0.101, 0.111] exhibited the narrowest 95% CIs, while self-esteem [0.037, 0.043], social awareness [0.027, 0.033], and sports policy [0.019, 0.024] had relatively wider CIs.

**Table 3:** Integrated weighting and ranking of evaluation indicators.

Indicators	Delphi weighting			Entropy weighting			Integrated weighting		
	Value	95%CI	Ranking	Value	95%CI	Ranking	Value	95%CI	Ranking
One-level indicators									
A: MA	0.215	[0.182, 0.246]	1	0.164	[0.111, 0.216]	3	0.212	[0.187, 0.237]	2
B: PH	0.203	[0.172, 0.236]	2	0.157	[0.146, 0.168]	4	0.191	[0.168, 0.214]	3
C: MH	0.188	[0.160, 0.225]	3	0.189	[0.176, 0.202]	2	0.213	[0.212, 0.214]	1
D: SEC	0.127	[0.109, 0.157]	5	0.206	[0.194, 0.218]	1	0.156	[0.117, 0.196]	4
E: MHE	0.125	[0.102, 0.155]	6	0.146	[0.134, 0.158]	5	0.109	[0.099, 0.120]	6
F: MHB	0.142	[0.115, 0.172]	4	0.138	[0.130, 0.146]	6	0.118	[0.116, 0.120]	5
Two-level indicators									
A1: speed	0.046	[0.043, 0.049]	5	0.032	[0.028, 0.036]	19	0.034	[0.014, 0.053]	6
A2: strength	0.038	[0.034, 0.042]	9	0.034	[0.029, 0.039]	14	0.029	[0.023, 0.034]	11
A3: CA	0.052	[0.049, 0.055]	3	0.039	[0.033, 0.045]	10	0.046	[0.028, 0.064]	3
A4: flexibility	0.032	[0.029, 0.035]	15	0.030	[0.025, 0.035]	22	0.021	[0.018, 0.023]	19
A5: balance	0.048	[0.045, 0.051]	4	0.029	[0.024, 0.034]	23	0.032	[0.005, 0.058]	8
B1: body shape	0.097	[0.092, 0.103]	2	0.068	[0.058, 0.078]	2	0.150	[0.109, 0.190]	2
B2: PP	0.106	[0.101, 0.111]	1	0.089	[0.076, 0.102]	1	0.215	[0.191, 0.238]	1
C1: EH	0.045	[0.042, 0.049]	6	0.033	[0.028, 0.038]	18	0.034	[0.017, 0.050]	7
C2: self-esteem	0.040	[0.037, 0.043]	8	0.044	[0.037, 0.051]	5	0.040	[0.034, 0.045]	4
C3: families	0.035	[0.032, 0.037]	11	0.045	[0.038, 0.052]	3	0.036	[0.022, 0.049]	5
C4: SC	0.034	[0.032, 0.037]	13	0.034	[0.029, 0.039]	12	0.026	[0.026, 0.026]	14
C5: schools	0.034	[0.031, 0.037]	12	0.033	[0.028, 0.038]	16	0.025	[0.023, 0.026]	16
D1: SO	0.030	[0.027, 0.033]	17	0.044	[0.037, 0.051]	4	0.031	[0.011, 0.050]	10
D2: RS	0.028	[0.025, 0.031]	19	0.041	[0.035, 0.047]	7	0.026	[0.008, 0.044]	15
D3: DM	0.024	[0.022, 0.026]	21	0.042	[0.036, 0.048]	6	0.022	[0.001, 0.046]	18
D4: SA	0.023	[0.021, 0.025]	24	0.040	[0.034, 0.046]	8	0.020	[0.002, 0.043]	21
D5: SM	0.023	[0.021, 0.025]	23	0.039	[0.033, 0.045]	9	0.020	[0.001, 0.042]	22
E1: SP	0.022	[0.019, 0.024]	25	0.028	[0.024, 0.032]	25	0.014	[0.005, 0.022]	26
E2: CE	0.023	[0.021, 0.024]	22	0.029	[0.025, 0.034]	24	0.015	[0.006, 0.023]	25
E3: SI	0.022	[0.020, 0.023]	26	0.030	[0.025, 0.035]	20	0.015	[0.003, 0.026]	24
E4: SE	0.027	[0.024, 0.029]	20	0.028	[0.024, 0.032]	26	0.017	[0.015, 0.018]	23
E5: FE	0.030	[0.028, 0.031]	18	0.030	[0.025, 0.035]	21	0.021	[0.021, 0.021]	20
F1: MB	0.042	[0.039, 0.044]	7	0.033	[0.028, 0.038]	17	0.031	[0.018, 0.043]	9
F2: lifestyle	0.038	[0.035, 0.040]	10	0.033	[0.028, 0.038]	15	0.029	[0.022, 0.035]	12
F3: PB	0.032	[0.029, 0.034]	14	0.034	[0.029, 0.039]	13	0.025	[0.022, 0.027]	17
F4: adaptation	0.031	[0.028, 0.033]	16	0.038	[0.032, 0.044]	11	0.026	[0.016, 0.035]	13

Ej, entropy value of evaluation indicators; Dj, coefficient of variation; Wj, indicator weights; MA, motor ability; PH, physical health; MH, mental health, SEC, social and emotional competencies; MHE, motor health environment; MHB, motor health behavior; CA, coordination ability; PP, physiological perception; EH, emotional health; SC, social contacts; SO, social awareness; RS, relationship skills; DM, responsible decision making; SA, self-awareness; SM, self-management; SP, sports policies; CE, community environment; SI, sports institutions; SE, school environment; FE, family environment; MB, motor behavior; PB, psychological behavior; CI, confidence interval.

### 3.2 Entropy objective weighting method

Among various objective weighting methods, the entropy method is particularly suitable for this study because it directly quantifies the information content inherent in indicator data, aligning well with the practical need for assessment tools that have strong discriminatory power. Moreover, the entropy method is entirely data-driven, grounded in clear mathematical principles, and free from subjective influence; it therefore serves as an objective counterbalance to the subjective weights derived from the Delphi method. The concept of “entropy” originated in thermodynamics and was later introduced into information theory by C. E. Shannon, where it is called information entropy.

The core advantage of the entropy method is that it determines weights based on the degree of differentiation within the data. By relying on the intrinsic information of the dataset, it avoids the subjectivity inherent in expert-assigned weights, overcomes the limitations of purely judgment-based weighting, eliminates interference from human factors, and produces an objective and reliable evaluation of the system. In this study, data pre-processing included data cleaning and outlier handling. Outliers were detected using the box-plot method and removed when identified. For missing values, cases with a low missing rate (< 5%) were handled by deletion; when data retention was necessary, mean imputation was applied, with the specific approach explicitly documented. After confirming the integrity of the original dataset, the main computational steps of the entropy weight method were carried out as follows.

#### 3.2.1 Data standardization

First, the original data were standardized to eliminate scale differences and directional effects among indicators, yielding the standardized matrix *X*. Among common standardization techniques, this study employed min–max normalization (range standardization). The entropy method essentially measures the information conveyed by data variability. Range standardization preserves the inherent dispersion and ordinal structure of the original data without distortion, thereby providing the most reliable input for entropy calculation. This, in turn, ensures that the subsequent integration of subjective and objective weights is mathematically rigorous and interpretable, avoiding complications arising from negative values or shifted data centers. As a non-parametric method, range standardization does not require the data to follow a normal distribution, making it especially suitable for the diverse indicators used in this study.

Positive indicators were standardized using Formula 5:


$$\begin{array}{c} {\text{X}}_{\text{ij}}=\frac{{\text{X}}_{\text{ij}}-{\text{X}}_{\text{j}}\min}{{\text{X}}_{\text{j}}\max-{\text{X}}_{\text{j}}\min}+1 \end{array}$$ (5)

Reverse (negative) indicators were standardized using Formula 6:


$$\begin{array}{c} {\text{X}}_{\text{ij}}=\frac{{\text{X}}_{\text{j}}\max-{\text{X}}_{\text{ij}}}{{\text{X}}_{\text{j}}\max-{\text{X}}_{\text{j}}\min}+1 \end{array}$$ (6)

Where *i* = 1, 2, …, *n* denotes the sample index, and *j* = 1, 2, …, *m* represents the indicator index.

#### 3.2.2 Calculation of contribution proportion

The contribution proportion *P*_*ij*_ of the *i*-th sample under the *j-*th indicator was computed to form a new matrix, as shown in Formula 7:


$$\begin{array}{c} {\text{P}}_{\text{ij}}=\frac{{\text{X}}_{\text{ij}}}{\sum_{\text{i }=\;1}^{\text{n}}{\text{X}}_{\text{ij}}} \end{array}$$ (7)

#### 3.2.3 Calculation of entropy value

The entropy value *E*_*j*_ of the *j*-th indicator was calculated using Formula 8:


$$\begin{array}{c} {\text{E}}_{\text{j}}=-\text{K}\sum_{\text{i }=\;1}^{\text{m}}{\text{P}}_{\text{ij}}\text{ln}\left({\text{P}}_{\text{ij}}\right) \end{array}$$ (8)

Where 0 ≤ *E*_*j*_ ≤ 1, and K= 1/ln (*n*), with *n* representing the number of samples.

#### 3.2.4 Calculation of variation coefficient

Because *E*_*j*_ is inversely related to data variability, the variation coefficient *d*_*j*_ was defined to characterize the degree of dispersion. A larger *d*_*j*_ indicates greater information content and a stronger influence of the indicator. The coefficient is computed as shown in Formula 9:


$$\begin{array}{c} {\text{d}}_{\text{j}}\;=\;1-{\text{E}}_{\text{j}} \end{array}$$ (9)

#### 3.2.5 Calculation of indicator weights

The weight *W*_*j*_ of the *j*-th indicator was obtained via normalization, as shown in Formula 10:


$$\begin{array}{c} {\text{W}}_{\text{j}}=\frac{{\text{d}}_{\text{j}}}{\sum_{\text{j }=\;1}^{\text{n}}{\text{d}}_{\text{j}}} \end{array}$$ (10)

The calculated weights for primary and secondary indicators, as well as the combined weights of secondary indicators, are presented in Table 4. The entropy-based weights for primary indicators are: motor ability (0.164), physical health (0.157), mental health (0.189), social and emotional competence (0.206), motor health environment (0.146), and motor health behavior (0.138). The entropy-based weights for secondary indicators are: motor ability-speed (0.196), strength (0.208), coordination (0.236), flexibility (0.181), balance (0.179); physical health-body composition (0.431), physiological perception (0.569); mental health-emotional health (0.173), self-esteem (0.231), family contact (0.239), social functioning (0.182), school role (0.176); social and emotional competence-social awareness (0.216), relationship skills (0.198), responsible decision-making (0.204), self-awareness (0.192), self-management (0.190); motor health environment-sports policy (0.195), community environment (0.196), physical training institutions (0.208), school environment (0.194), family environment (0.207); and motor health behavior-sport behavior (0.237), lifestyle (0.242), psychological behavior (0.248), social adaptation (0.273). The combined weights of secondary indicators were computed using the product method, ranging from 0.028 to 0.089; specific values and rankings are provided in Table 4.

**Table 4:** Entropy method indicator weights and combined weights.

Single Weight	A: MA	B: PH	C: MH	D: SEC	E: MHE	F: MH	*E_*j*_*	*D_*j*_*	*W_*j*_*	Composite Weight	Ranking
	0.164	0.157	0.189	0.206	0.146	0.138					
A1: speed	0.196						0.963	0.037	0.196	**0.032**	19
A2: strength	0.208						0.960	0.040	0.208	**0.034**	14
A3: CA	0.236						0.955	0.045	0.236	**0.039**	10
A4: flexibility	0.181						0.966	0.034	0.181	**0.030**	22
A5: balance	0.179						0.966	0.034	0.179	**0.029**	23
B1: body shape		0.431					0.961	0.039	0.431	**0.068**	2
B2: PP		0.569					0.948	0.052	0.569	**0.089**	1
C1: EH			0.173				0.966	0.034	0.173	**0.033**	18
C2: self-esteem			0.231				0.954	0.046	0.231	**0.044**	5
C3: families			0.239				0.953	0.047	0.239	**0.045**	3
C4: SC			0.182				0.964	0.036	0.182	**0.034**	12
C5: schools			0.176				0.965	0.035	0.176	**0.033**	16
D1: SO				0.216			0.948	0.052	0.216	**0.044**	4
D2: RS				0.198			0.953	0.047	0.198	**0.041**	7
D3: DM				0.204			0.951	0.049	0.204	**0.042**	6
D4: SA				0.192			0.954	0.046	0.192	**0.040**	8
D5: SM				0.190			0.955	0.045	0.190	**0.039**	9
E1: SP					0.195		0.965	0.035	0.195	**0.028**	25
E2: CE					0.196		0.965	0.035	0.196	**0.029**	24
E3: SI					0.208		0.963	0.037	0.208	**0.030**	20
E4: SE					0.194		0.966	0.034	0.194	**0.028**	26
E5: FE					0.207		0.963	0.037	0.207	**0.030**	21
F1: MB						0.237	0.966	0.034	0.237	**0.033**	17
F2: lifestyle						0.242	0.965	0.035	0.242	**0.033**	15
F3: PB						0.248	0.964	0.036	0.248	**0.034**	13
F4: adaptation						0.273	0.961	0.039	0.273	**0.038**	11

Ej, entropy value of evaluation indicators; Dj, coefficient of variation; Wj, indicator weights; MA, motor ability; PH, physical health; MH, mental health, SEC, social and emotional competencies; MHE, motor health environment; MHB, motor health behavior; CA, coordination ability; PP, physiological perception; EH, emotional health; SC, social contacts; SO, social awareness; RS, relationship skills; DM, responsible decision making; SA, self-awareness; SM, self-management; SP, sports policies; CE, community environment; SI, sports institutions; SE, school environment; FE, family environment; MB, motor behavior; PB, psychological behavior. The bolded values represent the combined weight results.

In the entropy weighting method, 95% confidence intervals (CIs) for the primary indicators were estimated using the t-distribution method, as shown in Table 3. Motor ability exhibited a relatively wide 95% CI [0.111, 0.216], while all other primary indicators were more stable. For secondary indicators, 95% CIs were computed using an approximation method. Among them, indicators including sports policy, balance, and community environment had the narrowest 95% CIs, while physiological perception, body composition, and social awareness had the widest intervals.

### 3.3 Subjective-objective integrated weighting method

Due to the dual nature of motor health assessment—encompassing both normative value judgments and objective statistical differentiation—purely subjective (e.g., Delphi) and purely objective (e.g., entropy) weighting approaches both have inherent limitations. The integrated weighting method aims to bridge the gap between the “ought-to-be” (normative expectations) and the “is” (empirical reality), thereby generating a more balanced weighting system that aligns theoretical guidance with practical discriminatory ability. This demonstrates the advantage of a mixed-methods research design. To achieve the integration of subjective and objective weighting, we constructed a combined weighting model that incorporates qualitative-subjective and quantitative-objective approaches. The objective method is used to adjust the weights obtained from the subjective method, which mitigates the arbitrariness inherent in expert judgments and yields final weights that better reflect empirical conditions. The integrated weights are calculated by combining the results of the two methods, as shown in Formula 11:


$$\begin{array}{c} {\text{W}}_{\text{j}}^{*}=\frac{{\text{W}}_{\text{j}}^{\left(1\right)}\cdot {\text{W}}_{\text{j}}^{\left(2\right)}}{\sum_{\text{i }=\;1}^{\text{n}}{\text{W}}_{\text{j}}^{\left(1\right)}\cdot {\text{W}}_{\text{j}}^{\left(2\right)}} \end{array}$$ (11)

In this formula, ${\text{W}}_{\text{j}}^{*}$ is the integrated weight, ${\text{W}}_{\text{j}}^{\left(1\right)}$ denotes the Delphi subjective weight of the *j-*th indicator, and ${\text{W}}_{\text{j}}^{\left(2\right)}$ denotes the entropy objective weight of the *j-*th indicator.

The integrated weights and their rankings are summarized in Table 3. The total combined weight for the six primary indicators is 1.0, with individual weights ranging from 0.109 to 0.213. The corresponding weights for the 26 secondary indicators, which range from 0.014 to 0.215, are also detailed in the table. The integrated weights for the primary indicators are as follows: motor ability (0.212), physical health (0.191), mental health (0.213), social and emotional competence (0.156), motor health environment (0.109), and motor health behavior (0.118). The integrated weights for the secondary indicators are: motor ability—speed (0.034), strength (0.029), coordination (0.046), flexibility (0.021), balance (0.032); physical health—body composition (0.150), physiological perception (0.215); mental health—emotional health (0.034), self-esteem (0.040), family contact (0.036), social functioning (0.026), school role (0.025); social and emotional competence—social awareness (0.031), relationship skills (0.026), responsible decision-making (0.022), self-awareness (0.020), self-management (0.020); motor health environment—sports policy (0.014), community environment (0.015), physical training institutions (0.015), school environment (0.017), family environment (0.021); and motor health behavior—sport behavior (0.031), lifestyle (0.029), psychological behavior (0.025), social adaptation (0.026).

In the integrated weighting method, 95% confidence intervals (CIs) for each weight were estimated using an approximate standard error method, as presented in Table 3. Among the primary indicators, mental health and motor-health environment had the narrowest 95% CIs, whereas social-emotional competence and physical health had the widest. Among the secondary indicators, social functioning, self-esteem, and community environment exhibited the narrowest 95% CIs, while responsible decision-making, body composition, and physiological perception had the widest.

### 3.4 Correlation analysis of different weighting methods

The subjective-objective integrated weighting approach represents not merely a superposition of methodologies, but a systematically constructed methodological framework that leverages the inherent logical complementarity between the two paradigms. It is designed to systematically integrate the strengths of subjective and objective weighting methods, thereby establishing a system that enhances the normative judgment characteristic of subjective methods while improving the objective statistical discrimination required for practical applications. To illustrate potential methodological divergences between subjective and objective weighting in practice, a Pearson correlation analysis was conducted to compare the results obtained from the three weighting approaches. The results are presented in Table 5.

**Table 5:** Pearson correlation analysis of three weighting methods.

Weighting methods	Integrated weighting	Delphi weighting	Entropy weighting
One-level indicators			
Integrated weighting	1		
Delphi weighting	0.771 (*P* = 0.072)	1	
Entropy weighting	0.600 (*P* = 0.208)	0.143 (*P* = 0.787)	1
Two-level indicators			
Integrated weighting	1		
Delphi weighting	0.917 (*P* = 0.000)^**^	1	
Entropy weighting	0.558 (*P* = 0.003)^**^	0.268 (*P* = 0.185)	1

^**^Significantly correlated at the 0.01 level (two-tailed).

Regarding the weighting of first-level indicators, no significant correlation was found between the comprehensive weighting method and the Delphi method (*r* = 0.771, *P* = 0.072 > 0.05, two-tailed), nor between the comprehensive weighting method and the entropy weight method (*r* = 0.600, *P* = 0.208 > 0.05, two-tailed). Similarly, the Delphi method and the entropy weight method showed no significant correlation (*r* = 0.143, *P* = 0.787 > 0.05, two-tailed). In contrast, for the second-level indicators, the comprehensive weighting method demonstrated a strong correlation with both the Delphi method (*r* = 0.917, *P* < 0.001, two-tailed) and the entropy weight method (*r* = 0.558, *P* = 0.003 < 0.01, two-tailed). However, no significant correlation was observed between the Delphi method and the entropy weight method (*r* = 0.268, *P* = 0.185 > 0.05, two-tailed).

Correlation analysis of the weights derived from distinct methods reveals that the integrated subjective–objective weighting approach is not a mere mechanical aggregation, but a systematic framework underpinned by the intrinsic complementarity of the two paradigms. This approach synergizes the strengths of both perspectives, establishing a robust mechanism that reconciles the normative insights of subjective valuation with the empirical discrimination inherent in objective data.

## 4 Discussion

Weighting methods in multi-criteria decision analysis are typically classified into three categories: subjective, objective, and integrated subjective-objective approaches. Even subtle variations in attribute weights can produce divergent outcomes, and the choice between “objective” and “subjective” methods significantly influences decision results (29).

Subjective weighting methods determine indicator weights based on experts' perceived importance of each criterion. For complex, multifaceted problems, group-based subjective methods are often employed to rank and measure criteria (30). Although widely used, subjective weighting can be problematic because it requires specialized expertise to accurately assign importance values; consequently, objective weighting methods play a crucial role in multi-criteria decision analysis (31).

Objective weighting methods determine weights based on the degree of inter-indicator linkage or the informational content each attribute provides. For example, some studies have applied several distinct objective weighting methods to parameter weighting, noting significant correlations between the results and underscoring the importance of method selection in prioritization tasks (32). However, objective methods also have limitations, and their results must be interpreted within practical contexts and informed by expert judgment (33). A key challenge is that subjective weights may overlook intrinsic data patterns, whereas objective methods can neglect expert intentionality, potentially yielding weights that do not align with decision-makers' contextual preferences or situational understanding.

Integrated Subjective–Objective Methods aim to balance decision-maker preferences with the need to reduce arbitrariness, thereby unifying subjective and objective inputs. A sound approach should simultaneously consider both the internal patterns in the data and expert experience. Integrated weighting methods synthesize different weighting results to produce more reliable alternative rankings across varying levels of expertise and criteria, making the selection of an appropriate weighting strategy critical (34). Moreover, integrated methods are effective in handling ambiguous and uncertain information (35) and are used to minimize the uncertainties inherent in purely subjective approaches by capturing interactions among indicators within multi-criteria decision-making frameworks (36). The combination of the Delphi method and the entropy method allows for complementary integration of their strengths, effectively addressing the practical needs of assessing motor health in pre-school children.

Therefore, this study adopted a comprehensive weighting method that integrates the Delphi (subjective) and entropy (objective) approaches, which is well-suited for the holistic evaluation of children's motor health. The Delphi method elicits relatively consistent information, opinions, and insights through iterative independent judgments from multiple experts, ultimately generating stable and reliable conclusions (28, 37, 38). The entropy method calculates weights based on the degree of variation in observed data, leveraging inherent differences in information content to objectively quantify the importance of each criterion, thereby reducing the impact of subjectivity and human bias (39). Combining subjective expert judgment with entropy-based objective weighting adjustment improves the objectivity and accuracy of the final weight assignments (40). The determination of weights for evaluation indicators reflects the intrinsic relationship between the indicator system and the evaluation model, and is closely aligned with the practical context of children's motor health. Through systematic processing and refinement, this approach provides a theoretical reference and practical insights for motor health-related practice.

This study sought to scientifically determine the weight coefficients for pre-school children's motor health evaluation indicators. A comprehensive subjective–objective weighting method was adopted, integrating the Delphi technique with the entropy method to conduct a rigorous estimation of the indicator system. At the primary indicator level, the weights in descending order were: mental health (0.213), motor ability (0.212), physical health (0.191), social and emotional competence (0.156), motor health behavior (0.118), and motor health environment (0.109). At the secondary indicator level, the combined weights in descending order were: physiological perception (0.215), body composition (0.150), coordination (0.046), self-esteem (0.040), family contact (0.036), speed (0.034), emotional wellbeing (0.034), balance (0.032), motor behavior (0.031), social awareness (0.031), strength (0.029), lifestyle (0.029), social adaptation (0.026), social functioning (0.026), relationship skills (0.026), school role (0.025), psychological behavior (0.025), responsible decision-making (0.022), flexibility (0.021), family environment (0.021), self-awareness (0.020), self-management (0.020), school environment (0.017), physical training institution (0.015), community environment (0.015), and sports policy (0.014). These findings deepen the understanding of comprehensive evaluation for pre-school children's motor health. They indicate that such assessment relies on indicators with differentiated weights, and requires an interdisciplinary approach to reveal the coupling effects of multiple factors on motor health outcomes.

To validate the applicability of the evaluation indicators and their assigned weights, confirmatory factor analysis was employed to examine the structure of the indicator system. The research group further assessed the overall and multi-group structural equation evaluation models. All incremental fit indices met acceptable standards, and the model satisfied criteria for parsimonious fit. The preset theoretical model showed good fit indices: *p* > 0.05, RMSEA < 0.05, GFI > 0.90, and AGFI >0.90, supporting the null hypothesis. These results suggest that the constructed indicator-weight system possesses practical value, and the measurement tools, including the scales, have also been validated.

Regarding the weighting of first-level indicators, no significant correlation was found between the comprehensive weighting method and either the Delphi method or the entropy weight method. Conversely, for the second-level indicators, the comprehensive weighting method exhibited strong correlations with both the Delphi method and the entropy weight method. Notably, across both levels of indicators, no significant correlation was observed between the Delphi method and the entropy weight method. It is worth noting that relying solely on either a subjective or an objective weighting method can lead to substantially different weight allocations. To simultaneously enhance the normative judgment inherent in subjective approaches and improve the objective statistical discrimination required for practical application, this study adopted an integrated weighting method. This approach not only combines the strengths of both techniques but also mitigates their respective limitations, thereby enhancing the practical applicability of the research and corroborating the potential methodological divergence between subjective and objective weighting methods. The findings on the consistency and divergence of methodological outcomes indicate that when a study requires integrating both subjective and objective information, and aims to improve the acceptability of a balanced result, the combined subjective-objective weighting approach represents an optimal solution. It serves as a crucial link between subjective cognition and patterns in objective data.

These findings have important implications for advancing the comprehensive evaluation of motor health among pre-school children, enriching relevant theoretical frameworks and addressing the practical challenge of weight assignment in indicator systems. Motor health assessment for pre-school children remains a promising area for further investigation. Future studies should build on this work to further examine the rationality of indicator weighting, and employ longitudinal designs to test the predictive validity of this weighting system for relevant long-term outcomes, thereby deepening overall understanding in this field. It is also noteworthy that the wider 95% confidence intervals observed for some indicators reflect greater divergence in expert opinions, which warrants further verification in future research.

### 4.1 Strengths and limitations

The strengths of this study lie in the successfully developed indicator weights and their strong practical applicability. First, the integrated subjective–objective approach acts as an intermediate pathway that bridges subjective cognition and objective data patterns. Second, the research findings provide valuable guidance for pre-school institutions and practitioners to carry out evidence-based motor health education. Finally, the whole process of this study maintains strict quality control, especially in the selection of authoritative experts, motor ability assessment, and questionnaire distribution and collection, all of which guarantee the robustness of the research.

This study has several limitations. The weighting results may be influenced by variations among experts and differences in sample data, which could affect the magnitude and ranking of the derived weights. These limitations highlight the need for repeated validation in future research to further confirm the scientific soundness and stability of the findings. In terms of sampling, this study was limited to pre-school children from five kindergartens in Guizhou Province, western China. Future studies should expand the sampling scope to include multi-regional samples to examine the consistency of objective weighting patterns across different populations. Methodologically, the combination of Delphi and entropy methods adopted in this study represents one of many feasible schemes. Although its suitability has been verified, alternative combinations (e.g., AHP + CRITIC) could be explored in future research, and comparative analyses can be conducted to test the stability of weighting results. Moreover, the predictive validity of evaluation outcomes under different weighting schemes should be further examined using real-world cases or longitudinal empirical data. Additionally, while parent-reported measures are useful, they may also introduce potential reporting biases.

In summary, from a pragmatist epistemological perspective, this study contributes to advancing the field of motor health in pre-school children, though many questions remain open for further investigation. We encourage more scholars to engage in research in this area to jointly promote progress in pre-school children's motor health. The current findings are intended to provide a useful reference and impetus for future studies, which may expand the breadth and depth of inquiry across geographical, age-related, cultural, ethnic, and national dimensions.

## 5 Conclusions

By employing a subjective-objective integrated weighting method, this study established a weighting system for assessment indicators based on two foundations: the inherent patterns within the data and professional expert knowledge. The weights obtained through this dual-foundation approach therefore have stronger rationality and credibility, and can provide valuable reference for accurate screening, priority intervention and sensitivity improvement of motor health outcome evaluation of pre-school children.

## Data Availability

All data presented in this study are included within the manuscript. Due to ethical and privacy considerations, certain baseline data involving preschool children cannot be made publicly available. However, de-identified data may be obtained by researchers upon reasonable request to the corresponding author, subject to approval from the institutional ethics committee.
